# Supplementary material for: Are carbon emissions trading and green financial instruments synergistic? -Comprehensive quantitative research based on content analysis
Source: PLoS One. 2024 Mar 7;19(3):e0298601. doi: 10.1371/journal.pone.0298601 (PMC10919601; doi:10.1371/journal.pone.0298601)
Supplement: S1 Data — (ZIP) [file pone.0298601.s001.zip › Data/地级市Z_F工作报告.zip!/║╙─╧/╞╜╢Ñ╔╜╩╨/└┤╫╘╣1⁄2╓┌║┼í╛┬φ┐╦╩2╛▌═°í┐_2005.docx]

一、2004年工作回顾

2004年是我市“实施二次创业，打造经济强市”,全面建设小康社会的第一年。一年来,市人民政府在省委、省政府和市委的正确领导下,团结带领全市人民,抢抓机遇,加快发展,圆满完成了市八届人大一次会议确定的各项任务。

——经济总量跃上新台阶。初步核算,全市生产总值达470.7亿元,新增105亿元,增长15.6%,为1994年以来最高,总量和增速均居全省第6位。人均生产总值突破1000美元,达到9616元,增长15%。

——财政收支大幅增长。全市地方财政收入21.5亿元,增收7亿元,增长46.6%,其中一般预算收入19.96亿元,增长43.5%,居全省第2位;财政支出37.96亿元,增长32.3%,财政实力明显增强。

——工业经济速度效益同步提高。规模以上工业企业实现增加值143.8亿元,增长23%;综合经济效益指数128.2%,提高17个百分点;实现利税45.3亿元,增长57.5%,其中利润19.6亿元,增长96%,均创历史最高水平。

——农村经济取得新进展。农林牧渔业实现增加值60.9亿元,增长9%,增速比上年提高6.4个百分点。全年粮食总产161.3万吨,增长11.4%,是近年来又一个高产年。

——内外贸易快速发展。全市进出口总值2.99亿美元,增长64.5%,其中出口1.25亿美元,增长25.8%;社会消费品零售总额131.6亿元,扣除物价上涨因素增长10.9%。

——就业再就业工作成效显著。全市城镇新增就业岗位6.4万个,2.7万名下岗失业人员实现再就业,其中“4050”人员9217人,均超额完成预期目标。城镇登记失业率控制在3.4%。

——人民生活进一步改善。全市城镇居民人均可支配收入7631元,扣除物价上涨因素增长8.3%;农民人均纯收入2369元,扣除物价上涨因素增长7.8%,扭转了多年来低速徘徊的局面。

这些成就,标志着我市经济社会发展跃上了一个新的战略起点。一年来,我们坚持科学发展观,在宏观调控中谋求发展,着力实现经济社会全面协调可持续发展。回顾过去的一年,我们主要做了以下工作:

(一)狠抓重点项目建设,经济社会发展支撑能力显著增强

抓住国家有保有压调整投资重点的机遇,乘势而上,充分发挥资源优势,千方百计克服资金、用地等制约因素,强力推进符合国家产业政策的重点项目建设,建成和开工了一批对经济社会发展带动能力强的重点项目。年初安排的81个重点项目,完成投资42.3亿元,建成投产11个。全市新增发电装机27万千瓦、日供水能力10万吨、日污水处理能力15万吨、有效灌溉面积3.1万亩、高速公路通车里程52公里、国省干线公路53公里、县乡公路294公里。投资对经济增长的拉动作用明显增强,全年全社会固定资产投资113.7亿元,增长45.1%,其中城镇固定资产投资94.9亿元,增长60.2%,居全省第4位。投资自主增长机制初步形成,民间投资37.1亿元,增长1.2倍,占投资的比重由28.3%提高到39.1%。

(二)加快结构调整步伐,综合竞争力进一步提高

推进工业结构优化升级。坚持把工业结构调整作为经济工作的重中之重,贯彻到实施“工业强市”战略的全过程,着力发展三大产业集群,全面推进八大工业基地建设。全年安排重点工业项目46个,完成投资15.5亿元;实施更新改造项目29个,投入技改资金16亿元。平煤集团80型焦炉改造、叶县蓝光电厂一期、汇源三期、海明黑液资源化、虹剑焦化、西气东输管线钢材技改、天鹰轨道交通电器、姚孟粉煤灰煤矸石多孔烧结砖等项目建成投产。全市规模以上工业新产品产值27.6亿元,增长22.2%。加快推进煤炭、铝土矿等资源整合,强力推动资源向优势企业集中,支持骨干企业做大做强。平煤集团与武钢、宝钢集团开展了广泛的合资合作,整合了梨园、新峰、白庙3家地方煤矿,全年销售收入110亿元,成为全省4家销售收入超百亿元企业之一。平煤、舞钢、神马、姚电、天瑞、天鹰6家企业进入全省100户成长性较好的重点企业行列,完成增加值92.4亿元,增长19%,对全市规模以上工业增加值贡献率达61.8%,拉动规模以上工业增长12.5个百分点。

加快服务业发展。在积极发展传统服务业的同时,重点支持现代服务业发展。全市实现旅游总收入8.6亿元,增长18.3%;接待游客200万人次,增长24.5%。规范房地产开发秩序,城镇以上房地产开发投资4.9亿元,增长40.9%。积极发展物流业,交通运输、仓储、邮电通信业增加值达到32.3亿元,增长21.4%。稳步发展金融业,年末金融机构存款余额390.2亿元,增长24%,其中城乡居民储蓄274.5亿元,增长18.7%;贷款余额221.8亿元,增长5.5%。全市第三产业增加值达到136.3亿元,增长12.9%。

大力实施县域经济突破。把发展壮大县域经济作为事关全局的重点工作,按照“工业首位、民营主体、项目带动、城乡一体”的总体思路,大力发展县域工业和民营经济。6县市实现生产总值300.4亿元,增速均在12%以上,占全市GDP的比重达63.8%。完成城镇固定资产投资45.6亿元,增长1.2倍;规模以上工业实现增加值52亿元,增长25.8%。舞钢、汝州、宝丰、叶县、新华和卫东6个县(市、区)财政收入过亿元,其中舞钢市突破2亿元。全市规模以上非公有制企业实现增加值50.4亿元,增长28.1%,实现税金7.8亿元,增长43.9%。小城镇建设步伐加快,全市城镇化率达到33.5%,是近年来城镇化步伐最快的一年。

(三)高度重视“三农”问题,农业基础地位得到巩固

坚持把解决“三农”问题作为各项工作的重中之重,围绕粮食增产、农业增效、农民增收,突出“六大产业”,推进“四大结构调整”工程,农业产业化和现代化步伐明显加快。畜牧业产值53.1亿元,增长6.8%,占第一产业产值的48.6%。完成造林面积22.3万亩,其中新增经济林5.9万亩。加强农产品质量安全体系建设,35家企业通过省无公害农产品产地认证。安排500万元农业结构调整资金,鼓励龙头企业、专业基地、产地批发市场建设,2家企业被确定为省级重点龙头企业,改扩建农产品市场18个。争取农口项目24个,新增国家和省投资2389万元。认真落实国家“降点直补”政策,农民实际减负增收9769万元。实施“农村百万劳动力转移工程”,完成农村劳动力转移培训11万人,新增劳务输出7.8万人,输出总量达50.8万人,农民外出务工收入达25.4亿元。

(四)城市建设取得新进展,承载能力不断增强

光明路南延全线贯通,三座湛河桥已完成工程量的80%,高阳路二期、鹰城广场改造正在稳步实施,文园路步行桥、快速通道连接线及明珠广场、七彩虹游园等一批街头游园建成投入使用。旧城改造步伐加快,锦绣花园、明珠城市花园、东方竣景小区主体已完工,繁荣街改造工程已开工建设。中水回用、西气东输天然气支线工程等公用事业建设步伐加快。开展了市容环境综合整治活动,占道经营、垃圾围城等问题得到进一步整治。新城区起步区7.6平方公里的各项规划设计已全部完成,出让划拨土地3857亩,20.5公里的道路已建成通车,水、电、气、暖基础设施配套已大部分完成,新城区已具备大开发、大建设的基础条件。

(五)各项改革稳步推进,对外开放成效明显

以贯彻实施《行政许可法》为契机,进一步转变政府职能,加快服务型、责任型政府建设,基本完成了政府机构改革和乡镇机关人员分流工作,行政审批制度改革进一步深化。保留市直行政许可主体40个,第一批保留行政许可事项215项。继续深化国有企业改革,积极推进粮食流通体制、农村信用社等改革。平煤、舞钢、神马、姚电四家大型企业通过项目合作、资本引入等形式,实现了强强联合和产权多元化;154户国有中小工业企业已改制141家,剩余13家企业7家进入破产程序,6家正在进行破产或转让前的准备工作。已有10家企业的13所学校基本与企业分离,平煤集团32所学校的分离工作进入实质性操作阶段。国有粮食购销企业分流安置职工4413人。农村信用社完成了10家县级联社及所属122家基层社的清产核资工作,增资扩股2.4亿元,清收不良贷款1.3亿元。坚持实施开放带动战略,扩大对内对外开放。加强对招商引资工作的指导和协调,成功举办了杭州、上海招商会、“世刘会”、“世叶会”、宝丰(上海)推介会等招商引资活动。全年共引进区外资金28.5亿元,实际利用外资3443万美元,增长29%,新批外商投资企业17家。加大对高压开关、碳化硅等拳头出口产品的扶持力度,实行进出口权备案制度,32家企业获得进出口经营权。

(六)加大就业和社会保障工作力度,切实维护群众利益

认真落实就业再就业扶持政策,新增城镇就业人数为近年来最高。做好“两个确保”和城市“低保”工作,推进三条保障线的衔接。调高了行政事业单位人员工资、企业退休人员养老金标准和城镇居民最低生活保障标准。发放下岗职工基本生活保障金7254万元,企业离退休人员养老金3.56亿元,均做到了按时足额发放。为8.1万名低保对象发放低保金5010万元,实现了应保尽保;投入413万元对4.3万农村特困人口实施救助,初步建立了城乡救助体系。在全省率先创办了“爱心超市”。筹措5846万元资金用于农村道路建设,完成“村村通”工程329公里,140个行政村实现通油路。筹措1528万元,实施农村饮水和饮水安全工程65处。加大扶贫开发力度,3.3万人口脱贫和解决温饱。战胜了百年一遇的严重洪涝灾害,安排救灾资金1631万元,开仓借粮50万斤,重建倒房6708间。全市清理建筑领域拖欠工程款1.5亿元,清欠农民工工资2560万元。全面落实安全生产责任制,深入开展了煤矿、非煤矿山、道路交通等7个重点领域的安全生产专项整治,大力推行安全质量标准化,提高了重点行业、重点领域的安全生产基础水平。大力整顿和规范市场经济秩序,实施食品、药品放心工程,保障人民群众身体健康和饮食、用药安全。

(七)加快发展社会事业,精神文明和民主法制建设得到加强

大力实施科教兴平和人才强市战略,实施科技计划项目102项、资金780万元,争取省科技计划项目21项、资金108万元。5家企业、7种产品被认定为省级高新技术企业和高新技术产品。投资2122万元,改造中小学D级危房1.5万平方米。筹资1605万元,对市一中、市二中、市一高进行建设改造。全市普通高中升学率达68.4%。平顶山学院挂牌成立,实现了当年本科招生。全市普通高等教育在校学生达到3.5万人。蒲城店遗址、汝州张公巷瓷窑遗址考古发掘取得重要收获。疾病防控和医疗救助体系建设进展顺利,投资改造乡镇中心卫生院15所。继续搞好新型农村合作医疗试点,参合农民达37.7万人。对全市既往有偿供血者进行普查,建立了艾滋病患者防治救助体系,实行患者免费救治。成功举办了市六运会和第八届全民健身节。继续做好人口和计划生育工作,稳定低生育水平,人口自然增长率4.9‰。加强环境保护,整治环境违法企业107家,取缔小炼焦池900道,违法煤堆、煤泥堆437堆,固化矸石山6座。民族宗教、审计统计、新闻出版、广播电视、人防、史志等各项社会事业不断进步。

深入学习贯彻“三个代表”重要思想,大力弘扬焦裕禄精神、“红旗渠”精神和“愚公移山”精神,广泛开展向任长霞等先进典型的学习活动。选派干部开展驻村帮扶工作。深入开展“道德规范进万家、诚实守信万人行”活动。大力推进未成年人思想道德建设,集中开展了网吧、互联网等专项整治。扎实推进精神文明创建活动,文明景区、文明城市创建活动取得新成效。拥有创建全国文明村镇工作先进村镇2个、全国精神文明建设工作先进单位2个、全国创建文明行业先进单位3个、全国社区建设示范区1个、省级文明单位166个。宝丰县创建为国家卫生县城。严格执行人大及其常委会通过的决议和决定,自觉接受人大法律、工作监督和政协民主监督,办理人大代表议案、建议和政协委员提案489件,办复率达100%。坚持科学民主决策,强力推进依法行政,政府工作法制化水平进一步提高。以创建综治先进市为契机,大力推进治安防控体系建设,组织开展“命案侦破攻坚战”,取得了明显成效。组织市县两级干部下访,着力化解信访积案,省交办的180件信访案件全部办结。制定突发事件应急预案,完善了应急机制。廉政建设和反腐败斗争取得新成效。驻平部队、武警官兵在抗洪抢险、维护社会稳定、支持地方经济建设中作出了重要贡献。

各位代表,这些成绩的取得,是省委、省政府和市委正确领导的结果,是人大、政协积极支持的结果,是全市人民共同奋斗的结果。在此,我代表市人民政府,向工作在全市各条战线的广大工人、农民、知识分子、干部、人民解放军驻平部队、武警官兵和公安民警致以崇高的敬意!向关心、支持我市现代化建设的各界人士表示衷心的感谢!

在肯定成绩的同时,我们也清醒地看到,我市与先进地市差距仍然较大,经济社会发展中仍然存在着一些突出矛盾和问题。农业生产基础还不稳固,农民增收难度加大;非公有制经济占GDP的比重偏小,经济外向度较低;信贷资金、建设用地供应偏紧,影响了城建、工业项目的实施;城乡新增劳动力较多,就业压力增大;物价上涨幅度较高,部分低收入群众的生活受到影响;煤矿安全生产形势相当严峻,一些地方和部门安全生产意识不强,责任落实不到位,重特大事故接连发生,给人民群众生命财产造成了不可挽回的损失;一些领导干部作风浮漂、形式主义比较严重;经济发展环 境还不够宽松,一些结构性和体制性矛盾依然存在。对此,我们一定高度重视,在今后工作中认真加以解决。

二、突出经济建设中心,努力实现经济跨越式发展

今年经济工作的总体要求是:坚持以邓小平理论和“三个代表”重要思想为指导,以科学发展观统领全局,围绕“实施二次创业，打造经济强市”,牢牢把握发展第一要务,大力调整经济结构,更加重视“三农”问题,积极推进改革创新,转变经济增长方式,以八大工业基地为依托,加快建设新型能源和工业基地,进一步推动经济的跨越式发展。主要预期目标为:生产总值增长11%左右,全社会固定资产投资增长18%,地方财政一般预算收入增长12%,社会消费品零售总额增长11%,外贸出口增长15%,实际利用外商直接投资增长25%,居民消费价格总水平上涨4%,城镇居民人均可支配收入增长7%,农民人均纯收入增长5%,城镇新增就业岗位5.5万个,城镇登记失业率控制在4.5%以内,人口自然增长率控制在6‰以内,工业企业质量和效益指标进一步提高。

为实现上述目标,重点做好以下八项经济工作。

(一)继续实施项目带动战略,着力增强发展后劲

紧紧围绕建设新型能源和工业基地,加快重点产业发展,加大对基础产业、基础设施的投入,积极实施一批事关经济社会发展全局的重大建设项目。今年初步安排固定资产投资项目(总投资500万元以上)262个,其中亿元以上项目89个。当年预期完成投资151亿元,其中亿元以上项目完成投资129亿元。

继续抓好电力项目建设。年内要完成平煤集团坑口电厂二期项目;加快姚电四期2×60万千瓦机组扩建、平顶山电厂2×20万千瓦热电联产机组扩建和瑞平公司2×15万千瓦热电机组项目。开工建设220千伏蒲楼等4个变电站、110千伏代庄等3个变电站。全年力争开工建设电力项目装机容量达157.5万千瓦,新增装机容量21.9万千瓦。加快平顶山电厂二厂4×90万千瓦机组、华电汝州电厂4×90万千瓦机组、叶县蓝光电厂二期等项目的前期工作。加大主电网建设力度,全面完成农网三期改造工程。

加速壮大煤炭产业。加快新井新矿建设,依托平煤集团建设超亿吨特大型煤炭基地。加快平煤集团十一矿改扩建、宁庄矿、张村矿和动力煤洗配中心技改工程建设进度。完成平煤集团庇山矿、朝川矿年产60万吨机焦项目一期工程。开工建设煤炭采掘信息化示范项目和汝州万和70万吨捣固焦工程。新增煤炭生产能力45万吨,新开工规模达到60万吨左右。做好平煤集团100万吨焦炭、夏店煤矿等项目的前期工作。抓好煤矿安全改造和采煤沉陷区治理。

加快重点基础设施和水利项目建设。漯平高速公路平顶山段年内建成通车,加快平临高速公路境内段工程建设步伐,开工建设郑石、太澳两条高速公路境内段工程。实施国省干线公路项目28个,完成干线公路改建415.6公里;改建县乡公路300公里,实现110个行政村通油路。完成昭平台灌区节水续建六七期、白龟山水库除险加固、老虎洞等12座中小型水库除险加固等工程的年度建设任务,完成西气东输平顶山支线和第四水厂等项目,加快建设燕山水库。争取早日开工建设南水北调中线平顶山段工程。

继续落实有保有压、松紧适度的投资调控政策,对投资项目“保重点、保续建、保投产达效”。同时,要筛选、储备一批科技含量高、能够带动产业结构优化升级的重点项目,尤其是重点工业项目,进一步提高重点项目对全市经济的支撑带动作用。

(二)加快推进工业结构调整,着力转变经济增长方式

按照走新型工业化道路的要求,强力实施“工业强市”战略,加大工业结构调整力度,加快经济增长方式转变,以八大工业基地为依托,以重点企业为龙头,集中力量抓好优势产业,促进传统产业新型化和新兴产业规模化,努力建设新型能源和工业基地。

做大做强传统产业。把工业结构调整作为经济工作的重中之重,充分发挥煤、盐、铁、铝等资源优势,用高新技术和先进适用技术改造传统产业,延伸产业链条,开发高端产品,形成产业集聚,提升行业集中度和竞争力,实现产业结构优化升级。完成中加钢铁二期、神马集团新增10万吨尼龙66盐和2万吨连续聚合切片、天瑞集团日产5000吨水泥熟料生产线及铁路重载合金钢铸件、中盐皓龙新增60万吨盐、汇源公司10万吨离子膜烧碱和10万吨专用树脂等项目。开工建设舞钢公司100万吨中厚板生产线项目。

培育壮大新兴产业。大力推进新兴产业发展,支持企业在高新技术领域开发战略性产品,扶持一批从事应用技术、信息技术开发的新兴企业。加大对具有潜在优势的新材料及环保领域的高新技术产品的开发力度,实现从煤炭产业到非煤产业、从地下企业到地面企业、从初加工产品向高附加值产品的转变,努力扩大非煤产业、高新技术产业和高附加值、深加工产品在工业经济和工业产品中的比重,逐步扩大高新技术产业在生产总值中的比重。抓好神马集团高模低缩涤纶工业丝项目建设,做好平煤集团50万吨甲醇、10万吨煤焦油工程，天鹰集团80万至100万伏高压隔离开关，中材环保新型环保设备等项目的前期工作。抓紧神马集团安全气囊丝、芳纶帘子布产品的试验和项目前期工作。

强力推进大公司、大集团战略。省级财政将增加工业结构调整和高新技术产业化专项资金,积极引导信贷资金和社会资金,加快百户重点企业规划项目实施。我们要进一步加大工作力度,大力推进以我市6家大型企业为代表的大公司、大集团发展战略,支持企业深化改革、加强管理和技术创新。平煤集团要努力延伸煤电建(材)、煤焦化(碳素)和煤炭—气化—尿素、甲醇化工产品产业链,加大与下游产品生产企业的联合、兼并、重组力度,实现投资主体多元化,力争销售收入达到120亿元。舞钢、神马、天鹰、天瑞、姚电公司销售收入要力争分别达到62亿元、32亿元、20亿元、15亿元、13亿元。同时,从全市九大行业中按年度销售收入选择30户企业作为市级重点企业予以倾斜支持。加强工业经济运行调节,抓好煤炭、电力供需衔接和运力协调,规范成品油市场管理,努力缓解煤电油运供求矛盾。

努力转变经济增长方式。广泛开展资源节约和综合利用,大力发展循环经济,切实做到从节约资源中求发展,从保护环境中求发展,逐步构建节约型的产业结构和消费结构,建设节约型社会。继续加大对高耗能、高污染等生产能力落后企业的淘汰力度,积极推行清洁生产。加快实施煤炭、铝土矿等资源整合。支持和鼓励平煤集团采取收购、兼并、控股、联合等方式,实施煤炭企业间的并购。严格控制焦化项目。重点搞好煤矸石、粉煤灰、垃圾等综合开发利用,推进中水回用、机焦废气回收利用工作,推广应用先进技术,提高资源节约的整体水平。

(三)进一步加强“三农”工作,促进农业增产和农民增收

解决好“三农”问题仍然是各项工作的重中之重。要认真落实中央一号文件,加快农村经济结构调整,着力提高农业综合生产能力,进一步强化政策支持,加强农村基础设施建设,积极推进农业产业化和现代化,努力实现粮食增产、农业增效、农民增收,促进农村经济社会全面发展。

围绕“六大产业”,继续抓好“四大调整”。一是调整种植业结构,突出发展优质粮油生产和高效经济作物。稳定提高优质粮油和优质烟叶种植面积,抓好叶县国家优质专用粮食基地建设,加快特色农业基地建设。二是调整农业产业结构,突出发展畜牧业、林果业。继续推进优质畜产品生产加工基地建设,大力发展规模养殖和畜牧园区,加快龙头企业建设步伐,推进畜牧产业化经营。加快奶源基地建设,大力发展乳制品加工龙头企业。抓好畜产品质量监测和动物疫病防治工作。大力发展林业生产,继续抓好退耕还林和平原绿化。三是调整农村经济结构,突出发展二、三产业。走工业化、集约化经营的道路,增强农产品加工业的辐射带动能力。大力发展服务业和劳动密集型企业。四是调整农村劳动力结构,突出发展劳务产业。强力实施“农村百万劳动力转移工程”,切实做好农民工的转移、培训、服务和管理工作,加强劳务基地建设,全年农村劳动力转移新增10万人。

提高农业科技和产业化经营水平。加强农业技术推广体系建设,提高农机装备水平,加大良种良法的推广力度。做好农产品质量认证和检验检测工作,促进农产品的绿色安全无害化和标准化生产,为实施农产品市场准入制度创造条件。大力发展农业产业化经营,抓好特色农产品的规模化种植和专业化生产。集中使用农业结构调整资金,重点加大对农业产业化经营龙头企业的扶持力度,提高粮食及畜产品加工转化能力。规划建设一批县域综合农业生产资料市场、乡镇生产资料配送中心和网点,形成相对完善的农产品市场服务体系,切实解决秸秆利用、农产品销售难等问题。积极发展农民专业合作组织和行业协会,提高企业生产经营和农民的组织化程度。

加强农村基础设施建设。积极利用国债项目,加快农村沼气、节水灌溉、人畜饮水、乡村道路等“六小工程”建设。大力开展土地治理和中低产田改造。搞好农业综合开发和大型灌区配套建设,加强农田水利基础设施建设,新建沼气池5000座、旱地小水窖4000座,发展有效灌溉面积3万亩、旱涝保收田4万亩、节水灌溉面积5万亩,解决3.2万农村群众饮水困难和1.4万群众饮水安全问题。

(四)搞好城市建设与管理,加快小城镇建设步伐

严格落实城市总体规划。城市总体规划已经修编完毕,要抓紧进行上报审批工作。强化规划实施监督,坚决维护城市规划的严肃性和权威性。进一步完善以总体规划为指导,专业规划、详细规划配套协调的城市规划体系,着手编制近期建设规划,完成绿地系统规划,加快编制市区管网综合规划和都市村庄及城乡接合部规划。及时编制新城区起步区以西发展规划。

强力推进新城区建设。新城区开发建设是我市城市建设的新亮点,也是我市经济发展的新高地。要加大起步区内的基础设施和重点项目建设力度。市政大厦和湖光小区要确保6月底投入使用,会议中心、深圳朗曼“0375首府”房地产、香港建业“森林半岛”房地产、上海懿龙“青山绿水”四星级宾馆、台湾伟立纺织品项目等一批重点项目要在5月底前全部开工建设,市政中心文化公园、湖滨大道及生态景观带、纬二路、经三路、污水处理以及水、电、气、暖、弱电等基础设施建设要加快步伐,确保年内配套到位。法院审判大厅、建委检测中心、市疾病控制中心、交通大厦、财政大厦、燃气大厦等17个项目要抓紧开工和建设,力争早出形象。加大融资力度,强化产业支撑,加快发展步伐,为实现“三年出形象、五年成规模、十年铸精品”的目标打下坚实基础。

加快老城区改造步伐。完成高阳路二期续建、体育路中段、曙光街东段、大众路、劳动路中段、大乌路中段、湛南路东段、开源路北段维修改造工程,改善城市道路微循环。美化城市出入口,完成东出入口绿化工程。尽快实施湛河三桥引线工程,确保三桥竣工即时发挥效益。开工建设市污水处理二期工程。加快生态园一期、文化宫联盟路连片开发与繁荣街、和平路商业步行街改造步伐,推进大营、马庄、西高皇等都市村庄改造工作,改善城市人居环境。重视高新区的发展,加强基础设施建设,强化载体和服务功能,优化东部城区功能,拓展东部发展空间。

加强城市管理与经营。结合创建国家卫生城市、优秀旅游城市、园林城市和省级文明城市活动,充分发挥三区城市建管作用,着力解决城市卫生、占道经营、垃圾围城、交通秩序,特别是背街小巷、城乡接合部脏乱差等人民群众反映强烈的突出问题,不断提高城市管理水平。盘活城市存量资产,争取金融资金,吸引民间资本,加大投入力度,加快城市建设,逐步实现由单纯靠财政资金搞建设,向全民参与、全社会投入城市建设的转变。特别要充分利用好新建城市道路、新城区建设拉动升值的土地,依法经营,公开拍卖,为城市基础设施建设筹措资金。进一步加快小城镇建设步伐。坚持规划先行、分类指导、分步推进的原则,以城关镇和中心集镇为重点,增强城镇的聚集效应和辐射效应,促进农村人口、生产要素向城镇集中,努力使城镇成为区域经济的发展平台,提高农村城镇化水平。大力支持争创文明县城、园林县城、生态县城、卫生县城工作,重点建设一批产业优势突出、各具特色的建制镇。省对重点县市拨付的城镇化建设引导资金已提前一年到位,要切实加强管理,确保资金使用效益。

(五)进一步扩大消费需求,加快发展第三产业

增加城乡居民收入,扩大消费需求。要把适当提高消费率作为经济发展的一项重要任务,逐步改变高投资、低消费的经济发展格局。要多渠道增加居民收入,加快社会保障制度建设,稳定居民收支预期,提高消费者信心。严密监测农业生产资料价格的上涨,降低农业生产成本。着力培育消费热点,促进消费结构优化升级。完善市场经济秩序,加强消费者权益保护,改善居民消费环境。

加快发展第三产业,培育新的经济增长点。进一步放宽政策,放开经营范围,鼓励各种经济成分的资金、技术、设备、房产、信息、劳务等投入第三产业,逐步提高第三产业在生产总值中的比重。完善促进服务业发展的政策措施,用先进理念和现代技术提升服务业水平。加强市场体系建设,进一步深化服务业各领域的产业分工,引导服务业向专业化方向发展。优先发展现代物流业,整合重组物流资源,培育现代大型物流集团。

积极发展文化、体育等需求潜力大的行业。加快发展现代金融、信息服务、电子商务等新兴服务业和咨询、法律服务、科技服务等中介服务业。

围绕全国优秀旅游城市创建活动,做大做强旅游业。要综合开发旅游资源,努力把旅游业培育成为我市国民经济的支柱产业。今年,要完成全市旅游总体规划编制工作,建立完善旅游资源和旅游项目数据库。整合旅游资源,加大旅游投入,高品位实施景区建设,打造旅游精品景区。强力做好石人山景区宣传推介工作,加大香山寺的保护开发力度。继续开展创建A级旅游景区工作,确保石漫滩国家森林公园二郎山、昭平湖、叶县县衙景区进入国家2A级景区,力争风穴寺、三苏园进入国家3A级景区行列。

(六)不断深化经济体制改革,全面提高对外开放水平

大力推进经济体制改革。一要继续深化国有企业改革。继续以产权制度改革为核心,以股份制为主要形式,大力发展混合所有制经济。规范国有企业改革行为,认真解决改制企业的突出问题。今年争取使已改制的13家国有大中型工业企业全部实现股权多元化,并在主辅分离、辅业改制方面取得进展。对7家进入破产程序的国有中小工业企业,尽快破产终结;其他6家实行一企一策,加快改革步伐。以企业办学校分离工作为重点,扩大分离企业办社会职能工作成果。以落实出资人职责为中心,健全国有资产监管体系。二要继续推进专项改革。今年,国家继续对农业生产实施“三补一免”政策,同时,省委、省政府决定,今年起我省全部免除农业税。各级各部门一定要抓好落实,确保农民得到比上年更大的实惠。要积极稳妥地推进县乡公共财政制度改革,制止发生新的乡村不良债务,防止农民负担反弹。粮食流通体制改革要争取10月底完成企业改制和减员分流任务。农村信用社改革要切实做好增资扩股、清收不良贷款工作,确保第一批9家农村信用联社顺利取得央行票据资金。三要继续推进其他领域的改革。进一步深化投资体制改革,规范政府投资范围和行为,减少、规范投资审批,落实企业投资自主权。继续抓好交通、城建、公用事业、水利等建管体制改革和社会事业领域改革,力求取得新的进展。

进一步扩大对外开放。一要进一步扩大出口规模。完善鼓励外贸出口的政策支持体系,进一步调整出口商品结构,继续抓好高压开关、电收尘器、碳化硅等机电和高新技术产品出口。积极组织和支持30户企业开拓国际市场,保持外贸出口持续快速增长。积极推进进出口经营权备案登记制,争取今年再使100家企业获得进出口经营权。二要扩大对外经济技术合作。支持已获外经签约权的平煤集团、火电一公司和天鹰集团搞好对外工程承包,力争使更多具备条件的企业获得外经签约权。鼓励和组织符合外方要求的企业职工及农民出国从事劳务活动,大力支持具有比较优势的高科技、名牌产品生产企业加快“走出去”步伐。三要继续加大招商引资力度。充分发挥产业和资源优势,主动承接、吸纳发达地区、知名企业的产业转移和辐射。组织好“郑交会”、“乌洽会”、乡镇企业东西合作洽谈会、上海、厦门、深圳和省政府赴香港、美国旧金山等大型招商活动参会工作,巩固杭州、上海招商成果,抓紧落实签约项目,建立“长三角”地区稳定的招商平台。进一步拓宽招商引资领域,力争在商业、交通、城市公用设施等方面有新的突破。

(七)发展壮大县域经济,扩大非公有制经济规模

按照“工业首位、民营主体、项目带动、城乡一体”的总体思路,以工业化为主导,以非公有制经济为重点,发展壮大县域经济。要切实用足用活中央、省、市的各项政策和规定,从简化行政审批、加大财政支持、拓宽融资渠道、改进建设用地管理等方面赋予县(市、区)更大的自主权和决策权。继续探索并进一步明确各县(市、区)的功能定位,完善县域经济目标管理体系和县域经济发展工作推进机制,加强对其经济社会发展的宏观指导,鼓励和支持各县(市、区)突出发展重点,培育壮大各具特色的支柱产业。六县(市)力争在全省综合经济实力排序中位次前移,人均GDP、人均财政收入、农民人均纯收入等指标赶上或超过全省平均水平,工业增加值、非公有制经济增加值占县域GDP的比重明显提高。

加快非公有制经济发展。要认真落实政策,加强和改进政府服务和管理,建立健全社会化服务体系,为非公有制经济发展创造平等竞争、一视同仁的法制环境、政策环境和市场环境。进一步放宽市场准入,鼓励和支持非公有资本进入基础设施、垄断行业、公用事业以及法律未禁止的其他行业和领域。支持天瑞集团、中加钢铁、汇源公司、洁石建材、九头崖集团、银龙集团、巨龙淀粉等一批带动能力强、科技含量高和就业岗位多的企业加速规模扩张,尽快做大做强。大力实施中小企业培训、创业和融资信用担保三大工程,加快完善中小企业信用担保体系和政府资金支持体系,促进非公有制经济快速健康发展。

(八)进一步加强财政收支管理,努力做好金融工作

继续强力组织财政收入。严格依法治税、依率计征,堵塞跑、冒、滴、漏,努力做到应收尽收,确保税款及时足额入库。狠抓财源建设,积极培育新兴财源,重点培植骨干财源。强化对重点税源的征管,同时管好零星税源。认真开展税收秩序整顿工作,加大非税收入征管和稽查力度,努力增加政府可用财力。

继续强化支出管理。树立过紧日子思想,严格控制一般性支出,保证农业、教育、科技、工资、再就业、社会保障等法定和重点支出需要。继续调整支出结构,整合财政专项资金,集中财力支持县域经济、重点企业和社会事业发展。全面推行“乡财县管”,认真落实“三奖一补”政策,积极争取转移支付,缓解县乡财政困难。深化财政体制改革,完善部门预算、国库集中收付、政府采购和“收支两条线”管理等制度。严格控制编制,减少财政供给。

积极协调金融部门支持地方经济发展。各金融机构要把握信贷重点,加强银企合作,确保重点企业、重点项目建设的资金需求,进一步扩大对中小企业、民营企业和县域经济发展的贷款规模。加大金融支农力度,积极扩大农户小额贷款和联保贷款规模。加强信用体系建设,优化金融发展环境。

三、注重统筹协调,努力构建社会主义和谐社会

(一)实施科教兴平和人才强市战略,坚持走可持续发展之路，大力推进科技进步与创新

加强与大专院校、科研院所的合作,打造科技创新平台。加快科技成果转化步伐,抓好科技计划项目推荐申报工作。加大科技投入,完善科技奖励制度,建成市、县科技推广网站。成立市技术市场协会,制订民营科技企业发展规划纲要。搞好县(市)科技工作试点。加大对企业和农业的支持力度。注重对自主知识产权的开发、利用和保护,培育优势品牌。

坚持优先发展教育。加大农村教育投入,整合教育资源,改善办学条件,努力消除中小学D级危房。大力发展高中阶段教育,争创国家级、省级示范性高中。做好“普九”巩固提高工作,确保通过“普九”复验。落实“两免一补”政策,解决贫困学生辍学问题。发展中小学现代远程教育,推进教育信息化。加强教育教学改革,全面推进素质教育。支持平顶山工学院、平顶山学院继续改善办学条件,加强重点专业建设。大力发展民办教育、职业教育和成人教育,促进各级各类教育协调发展,积极构建终身教育体系。

大力实施人才强市战略。牢固树立人才是第一资源的观念,注重培养、吸纳、用好和稳定各类人才,建立整体推进人才资源开发的政策体系。完善人才培养、引进、使用的各项具体政策,营造尊重人才、培养人才、留住人才、鼓励创新的良好氛围。加大紧缺人才、高层次人才和国外智力引进工作力度,加强专业技术人员队伍建设。做好农村人才资源开发培养工程,变人口压力为人才资源。完善人才市场功能,为各类经济组织提供人才服务。

坚持可持续发展。依法加强环境保护和生态建设,以大气污染防治为重点,全面推进蓝天工程;突出水污染防治,提高城市居民饮用水质量;继续开展对不法排污企业的治理整顿,加大项目监管力度,加快环保基础设施建设。强化矿权管理,加强地质环境保护和地质灾害监测预防;深化国土资源有偿使用制度,大力开展矿产资源管理秩序整顿,提高资源的开发利用率。强力推进计划生育工作综合改革,做好基层基础工作,加强流动人口管理,搞好性别比专项治理、农村计划生育家庭小康工程,严格控制人口出生率,确保计划生育工作在全省的先进位次。

(二)加强精神文明建设,繁荣各项社会事业

全面贯彻落实《公民道德建设实施纲要》,深入开展社会公德、职业道德和家庭美德教育。进一步弘扬焦裕禄精神、红旗渠精神和愚公移山精神。加强未成年人思想道德建设和大学生思想政治教育。全面加大“四城”联创力度。深入开展群众性精神文明创建活动,继续抓好文明单位创建工作。加强诚信教育,倡导诚实守信的良好社会风气。加强国防教育,搞好国防动员、人民防空和民兵预备役建设,巩固双拥模范城成果。充分发挥工会、共青团、妇联等人民团体的桥梁纽带作用。巩固和扩大基层民主,继续大力推进厂务公开、村务公开,完善村民自治、社区民主管理和企业民主管理制度。做好第五届村民委员会换届工作。

实施文化精品战略,繁荣文学艺术、新闻出版、广播影视等各类文化事业。加快文化基础设施建设步伐,做好文物古迹的保护、利用和管理工作;大力推进基层文化建设,积极启动民间文化保护工程,依法加强文化市场的管理和整治。积极推进有线电视数字化和自然村广播电视“村村通”工程。切实抓好市第一人民医院病房楼、疾控中心、市传染病医院等重点项目建设;进一步健全和完善突发公共卫生事件应急机制、疾病防控体系和卫生执法监督体系;加大艾滋病等传染病防治工作力度,加快农村医疗卫生体系建设,大力发展城市社区卫生服务。广泛开展全民健身运动,提高竞技体育水平。推进统计改革和建设,认真做好第一次全国经济普查工作。继续做好民族宗教、盐业管理、史志、对台、侨务、档案、气象、残疾人、老龄等工作。

(三)做好就业再就业和社会保障工作,维护群众切身利益

进一步加大就业再就业工作力度。大力发展劳动密集型产业、就业容量大的非公有制经济和中小企业,努力开发社区服务和物业管理、养老托幼、家政服务、医疗保障、文化娱乐等社会性就业岗位。鼓励国有大中型企业实施主辅分离和辅业改制,拓宽富余人员的分流安置渠道。加大再就业资金投入,继续抓好小额贷款、税费减免等优惠政策的落实。健全再就业援助制度,搞好职业介绍、就业培训等再就业服务。引导下岗失业人员转变就业观念,鼓励下岗失业人员自谋职业和自主创业。大力开发公益性岗位,对“4050”人员等特殊就业困难群体实行就业援助。做好高校毕业生的就业指导服务和复员转业军人、城镇新增劳动力的安置工作。加大劳动保障监察执法力度,维护劳动者的合法权益。

切实做好社会保障工作。巩固“两个确保”,确保“两金”按时足额发放。积极稳妥地推进下岗职工基本生活保障向失业保险并轨,实行企业职工基本养老保险市级统筹。继续扩大城镇社会保险覆盖面,提高非公有制企业职工参保率,强化社会保险费的征缴、清欠和支出管理。强力推进医疗保险制度改革和工伤保险制度改革,搞好农村新型合作医疗制度试点工作。继续抓好低保政策的落实,做到动态管理下的应保尽保。切实保障妇女、未成年人、老年人、残疾人的合法权益,在全社会形成尊老助残的氛围。

认真解决关系人民群众切身利益的问题。进一步规范土地征用、城镇拆迁、企业改制工作,加大清理拖欠工程款和农民工工资力度,切实保护城乡居民的合法权益。做好受灾群众生产生活救济工作。认真实施“整村推进”扶贫计划,搞好定点帮扶,积极开展智力扶贫和科技扶贫,增强贫困地区的造血功能,尽快消除绝对贫困并巩固扶贫成果。逐步完善农村特困户生活救助制度,确保困难群众有饭吃、有衣穿、有房住、有病能得到及时医治,不让学生因家庭贫困而失学。继续整顿和规范市场经济秩序,严厉打击制售假冒伪劣食品、药品等违法犯罪活动,保护人民群众生命安全和身体健康。继续加大清理整顿“三会一部”工作力度。做好社会福利、社会救济、优抚安置和社会互助等工作,努力实现社会公平。

(四)坚持以人为本、安全第一,强化安全生产监督管理

全面落实安全生产责任制,突出企业主体责任,强化部门监督管理责任,落实各级领导责任,完善责任目标管理体系。要把煤矿安全生产作为安全工作的重中之重,贯彻落实《安全生产法》,严格执行“五个政府令”、“四个委派”、领导包矿和严防死守及事故责任追究制度。认真开展煤矿安全稽查活动,加大行业管理、执法监督力度,严厉打击违法违规生产行为。狠抓现场管理、技术管理和安全装备投入,强化全员培训,强力推进质量标准化,加快构建煤矿安全监管工作的长效机制。

进一步深化安全生产专项整治工作,突出抓好煤矿、非煤矿山、危险化学品、交通运输、公众聚集场所、民爆器材、烟花爆竹、燃气管道八项专项治理整顿工作,特别是做好煤炭资源整合期间的监管监控工作。继续抓好重点地区、重点行业、重点企业、重点场所、重点部位和重大危险源的安全监管工作,加大隐患排查、整改和监管监控力度。同时,认真做好消防安全、校园安全、防汛安全、森林防火等各项安全基础工作。

(五)高度重视信访稳定工作,打造“平安平顶山”

要把改革的力度、发展的速度和社会可承受程度统一起来,以维护人民群众利益为根本出发点和落脚点,在各项决策和实际工作中高度重视并切实维护群众利益。努力做好群众来信来访和人民内部矛盾纠纷排查调处工作,进一步完善信访制度,确保信访渠道畅通,力争把矛盾解决在基层、化解在萌芽状态。围绕创建综合治理先进市,深入开展以“命案攻坚”为龙头的“严打”整治斗争,严厉打击、严密防范各类刑事犯罪活动,坚决遏制和减少重大刑事案件的发生。防范和打击“法轮功”等邪教组织和境外敌对势力的渗透破坏活动。扎实推进社会治安防控体系建设,加强基层安全创建工作,形成覆盖城乡的治安防控网络,创建“平安社区”、“平安单位”、“平安村镇”,建设“平安平顶山”。

四、加强政府自身建设,提高依法行政能力

面对改革开放和现代化建设的新形势、新任务、新要求,各级政府必须牢固树立科学发展观和正确政绩观,按照科学执政、民主执政、依法执政对政府的要求,不断提高行政能力,努力建设勤政、廉洁、务实、高效的政府,建设全市人民满意的政府。

全面推进依法行政,建设法治政府。坚持依法治市,认真贯彻《行政许可法》,落实《全面推进依法行政实施纲要》,更新政府行政理念,完善行政程序,规范行政行为,健全行政监督机制,把依法行政贯穿于政府工作的全过程,努力做到合法行政、程序正当、高效便民、诚实守信、权责统一。要健全公众参与、专家论证和政府决策相结合的科学民主决策机制,完善行政决策程序,建立决策跟踪反馈和责任追究制度。继续推行行政执法责任制和执法过错追究制,认真解决有法不依、执法不公和多头执法、执法扰民等群众反映强烈的问题,把政府行为纳入法制化轨道,做到有权必有责、用权受监督、侵权要赔偿、违法要追究。要坚决贯彻市人大及其常委会各项决议和决定,坚持重大事项报告制度,自觉接受法律和工作监督。坚持重大问题决策前认真听取政协及各民主党派、工商联、无党派代表人士的意见,自觉接受民主监督。认真办理人大议案、建议和政协提案。进一步加强监察、审计等部门的专项监督和行政机关内部监督,自觉接受司法监督和社会监督。

转变政府职能,提高行政能力。在继续加强经济调节和市场监管的同时,更加重视政府的社会管理和公共服务职能,把精力和财力更多地放到推进社会发展和解决民生问题上来,把不该管、管不了、也管不好的事情进一步交给企业、市场和中介机构。增强贯彻落实科学发展观的自觉性和坚定性,着力提高五种能力:一是结合实际,创造性地贯彻执行党的路线方针政策的能力。二是以经济建设为中心,促进经济发展的能力。三是统筹兼顾,促进协调发展的能力。四是加强社会管理,搞好公共服务的能力。五是维护稳定,应对复杂局面和处理突发事件的能力。加强政府对国民经济和社会发展的规划管理,以科学发展观为指导,抓好“十一五”规划的编制工作。积极推进政府管理创新,合理确定部门职能。加强行政效能监察,严格执行工作责任制和责任追究制,确保各项决定不折不扣地得到执行,落到实处。加强电子政务建设,大力促进政府办公网络化、信息化,提高政府工作效率。

搞好政风建设,做到为政清廉。以“保持共产党员先进性教育活动”为契机,认真解决在思想、组织、作风以及工作方面存在的突出问题,使人民政府始终成为人民利益的忠实维护者,使广大公务员树立正确的理想信念和高尚的道德情操,永葆人民公仆的本色。要坚持实事求是,严格按照客观规律办事,多做打基础、管长远、惠百姓的工作,努力使各项工作经得起实践、历史和群众的检验;坚持群众路线,认真听取群众意见,自觉接受群众监督,充分调动人民群众的积极性和创造性,发挥人民群众在全市改革开放和现代化建设中的主体作用;坚持求真务实,说实话、办实事、求实效,力戒作风浮漂和推诿扯皮,把更多的时间和精力用在调查研究和工作落实上;坚持勤俭节约,反对铺张浪费,坚决制止不切实际、不计成本、不问效果的错误做法;坚持勤政廉政,积极推行政务公开,加大源头治理力度,切实解决人民群众反映强烈的热点、难点问题,进一步加强廉政建设和反腐败斗争,真正做到“为民、务实、清廉”。

各位代表,“实施二次创业，打造经济强市”,全面建设小康社会,是490万平顶山人民共同的奋斗目标。我们面临的任务艰巨而繁重,我们肩负的责任重大而光荣。让我们高举邓小平理论和“三个代表”重要思想伟大旗帜,在省委、省政府和市委的坚强领导下,抓住机遇,开拓创新,求真务实,埋头苦干,努力完成今年经济和社会发展的各项任务,为全面实现经济振兴、社会和谐、人民富足而努力奋斗！
